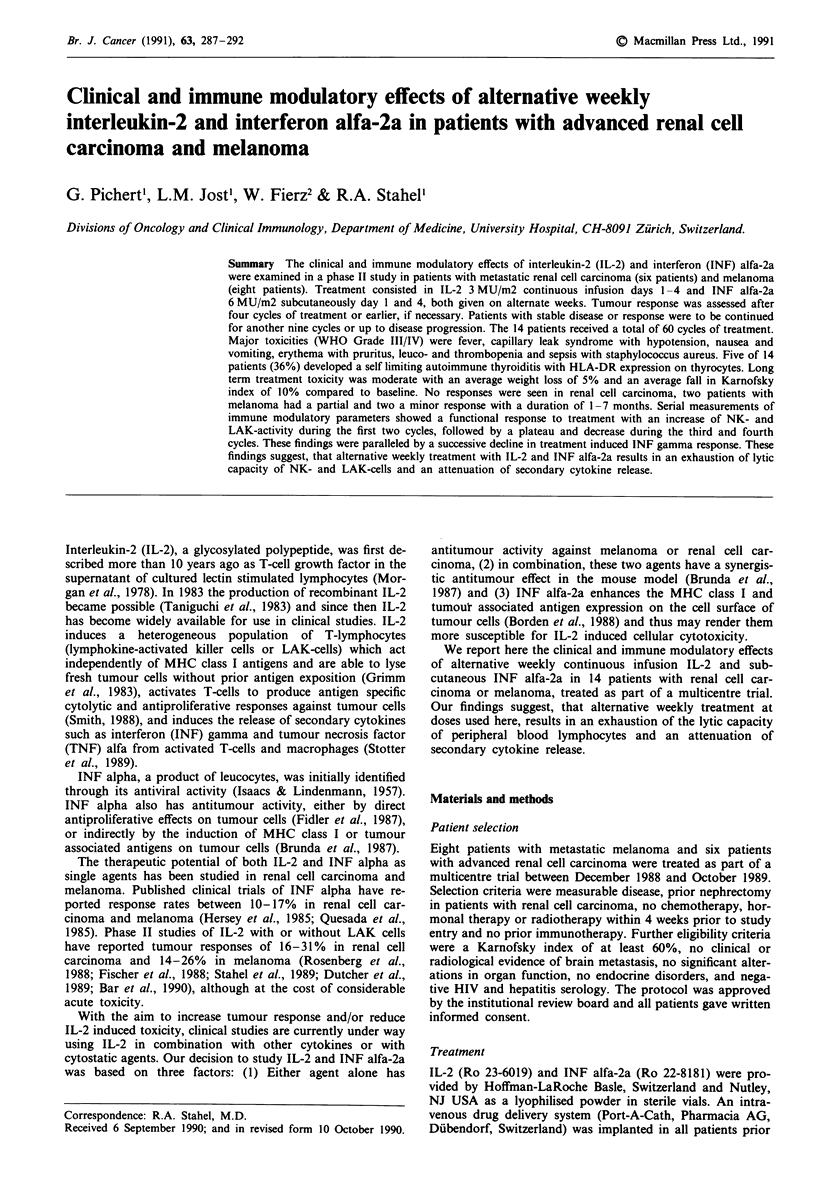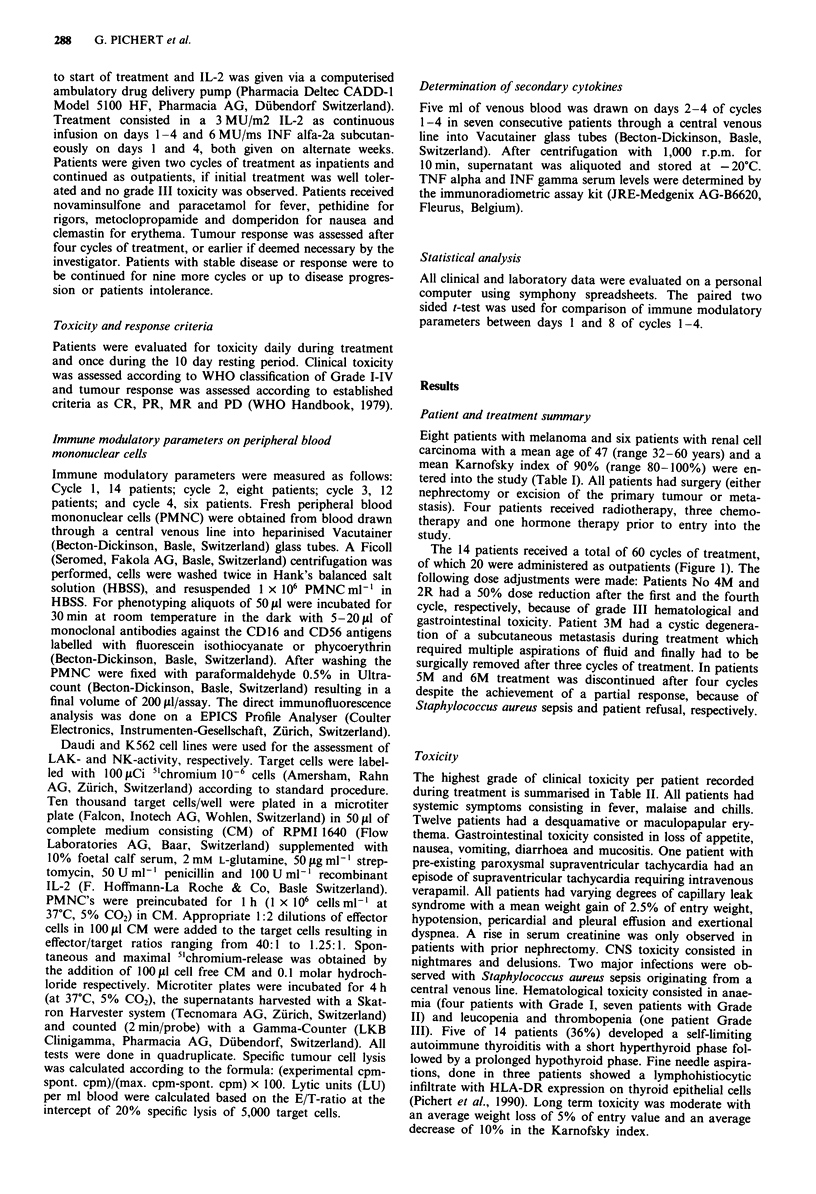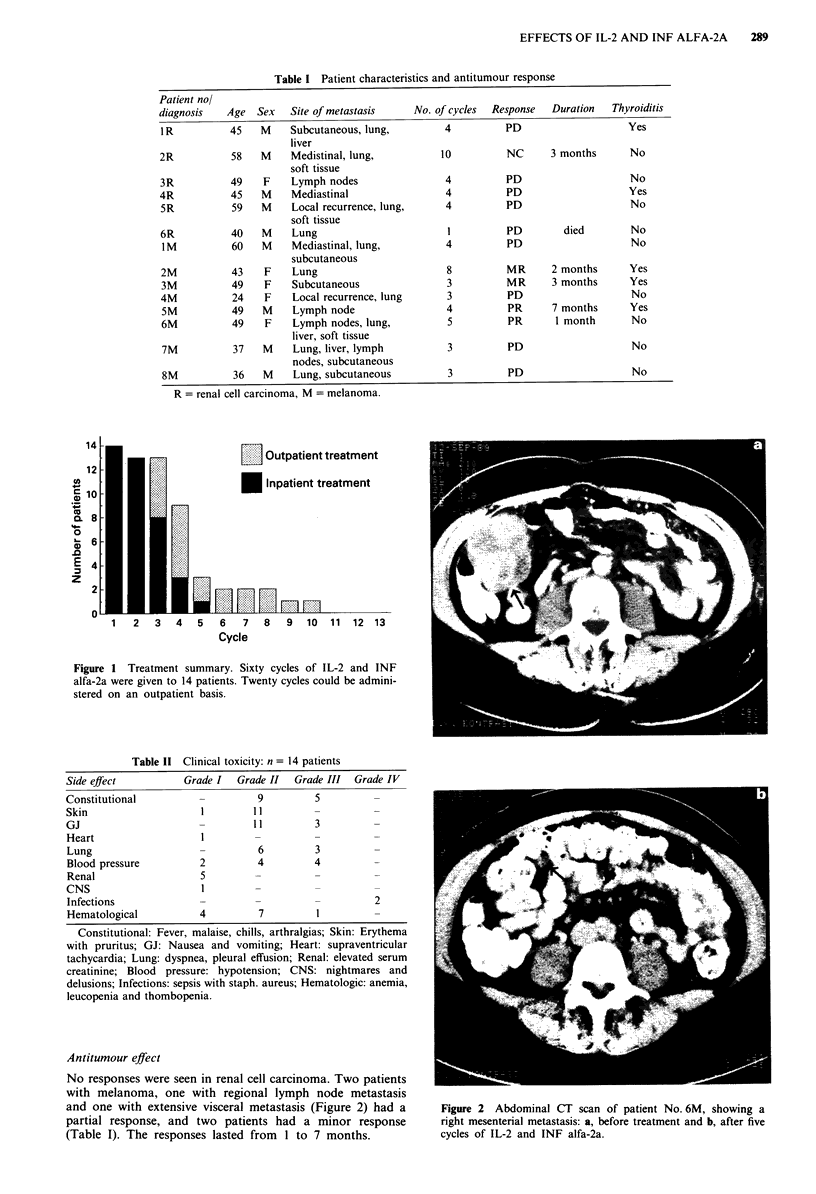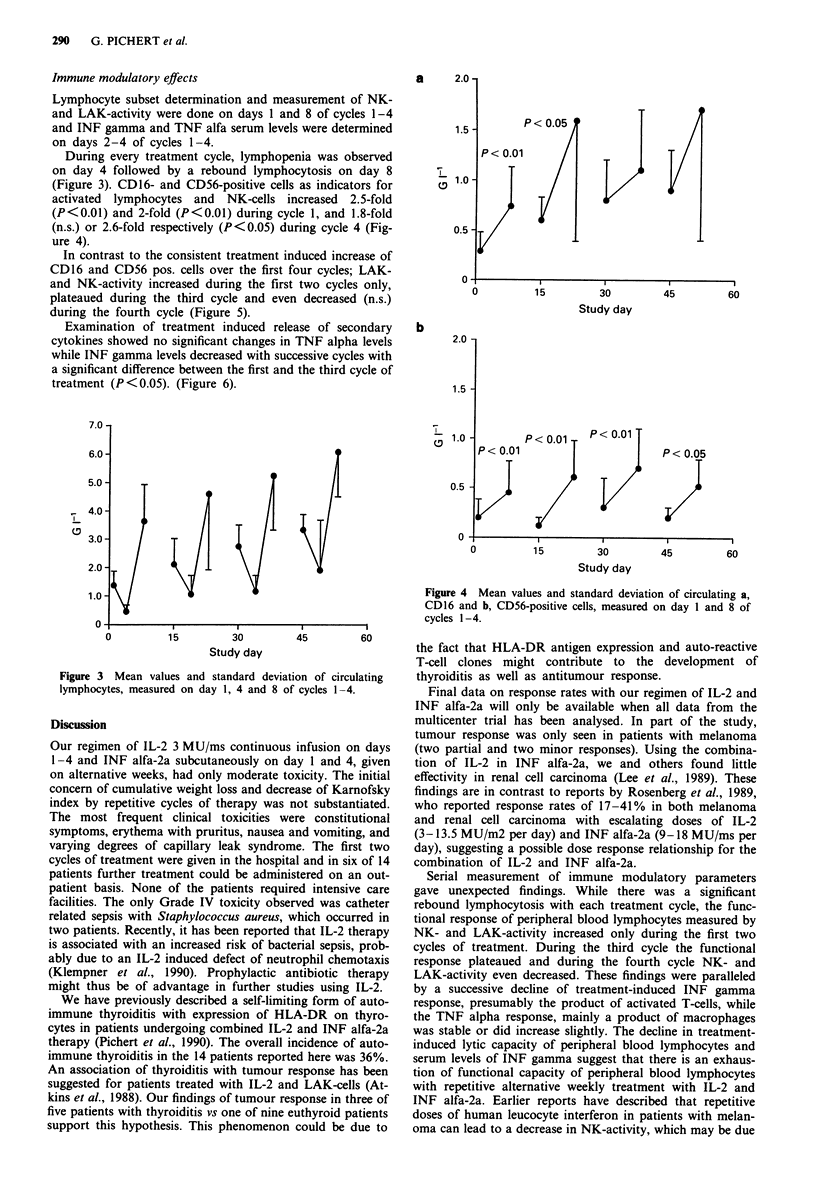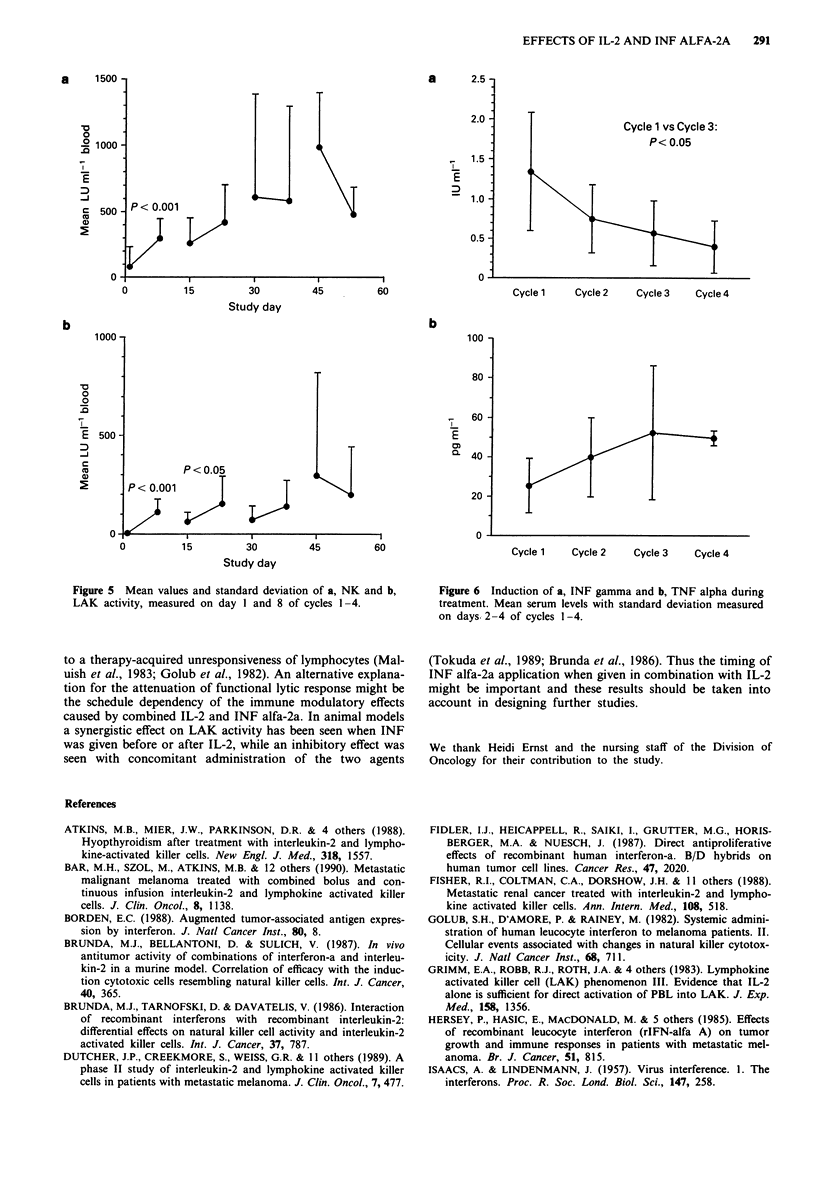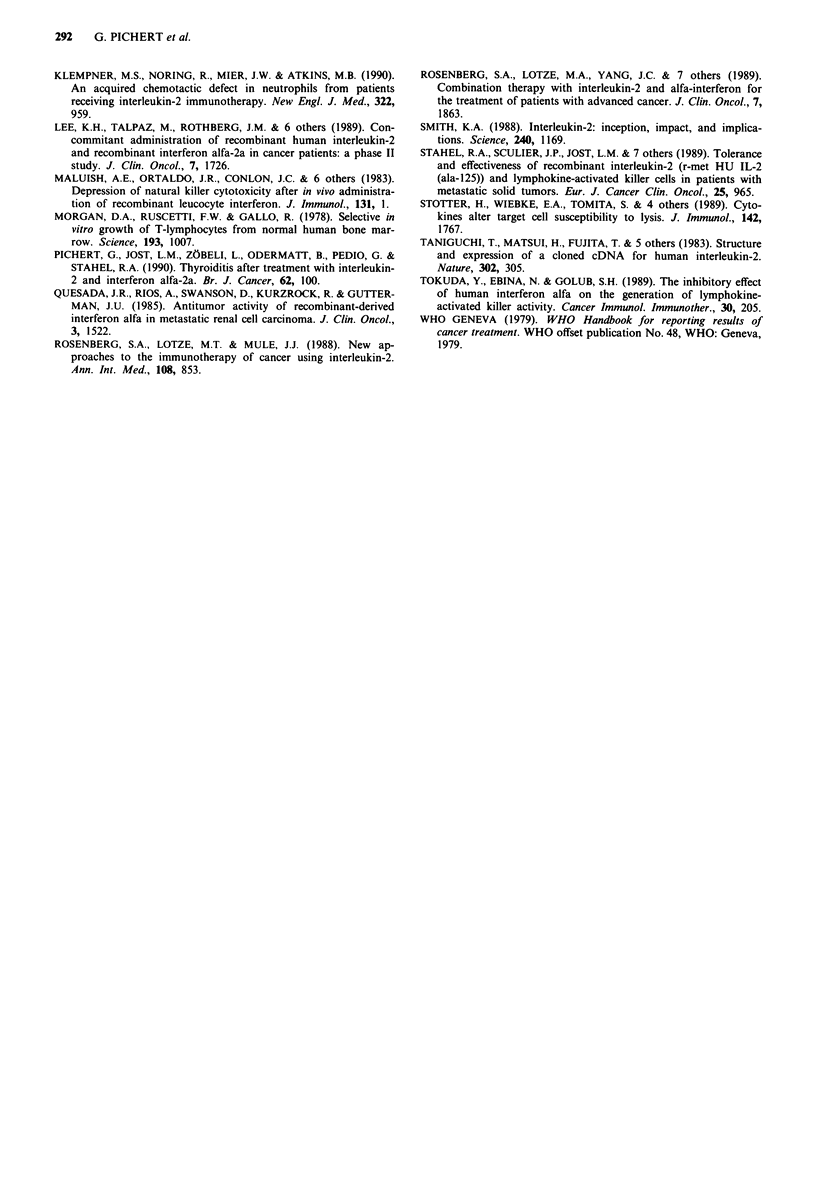# Clinical and immune modulatory effects of alternative weekly interleukin-2 and interferon alfa-2a in patients with advanced renal cell carcinoma and melanoma.

**DOI:** 10.1038/bjc.1991.67

**Published:** 1991-02

**Authors:** G. Pichert, L. M. Jost, W. Fierz, R. A. Stahel

**Affiliations:** Department of Medicine, University Hospital, Zürich, Switzerland.

## Abstract

**Images:**


Br. J. Cancer (1991), 63, 287-292 © Macmillan Press Ltd., 1991

### Clinical and immune modulatory effects of alternative weekly interleukin-2 and interferon alfa-2a in patients with advanced renal cell carcinoma and melanoma

G. Pichert[1], L.M. Jost[1], W. Fierz[2] & R.A. Stahel[1]

*Divisions of Oncology and Clinical Immunology, Department of Medicine, University Hospital, CH-8091 Zürich, Switzerland.*

**Summary** The clinical and immune modulatory effects of interleukin-2 (IL-2) and interferon (INF) alfa-2a were examined in a phase II study in patients with metastatic renal cell carcinoma (six patients) and melanoma (eight patients). Treatment consisted in IL-2 3 MU/m2 continuous infusion days 1-4 and INF alfa-2a 6 MU/m2 subcutaneously day 1 and 4, both given on alternate weeks. Tumour response was assessed after four cycles of treatment or earlier, if necessary. Patients with stable disease or response were to be continued for another nine cycles or up to disease progression. The 14 patients received a total of 60 cycles of treatment. Major toxicities (WHO Grade III/IV) were fever, capillary leak syndrome with hypotension, nausea and vomiting, erythema with pruritus, leuco- and thrombopenia and sepsis with staphylococcus aureus. Five of 14 patients (36%) developed a self limiting autoimmune thyroiditis with HLA-DR expression on thyrocytes. Long term treatment toxicity was moderate with an average weight loss of 5% and an average fall in Karnofsky index of 10% compared to baseline. No responses were seen in renal cell carcinoma, two patients with melanoma had a partial and two a minor response with a duration of 1-7 months. Serial measurements of immune modulatory parameters showed a functional response to treatment with an increase of NK- and LAK-activity during the first two cycles, followed by a plateau and decrease during the third and fourth cycles. These findings were paralleled by a successive decline in treatment induced INF gamma response. These findings suggest, that alternative weekly treatment with IL-2 and INF alfa-2a results in an exhaustion of lytic capacity of NK- and LAK-cells and an attenuation of secondary cytokine release.

Interleukin-2 (IL-2), a glycosylated polypeptide, was first described more than 10 years ago as T-cell growth factor in the supernatant of cultured lectin stimulated lymphocytes (Morgan *et al.*, 1978). In 1983 the production of recombinant IL-2 became possible (Taniguchi *et al.*, 1983) and since then IL-2 has become widely available for use in clinical studies. IL-2 induces a heterogeneous population of T-lymphocytes (lymphokine-activated killer cells or LAK-cells) which act independently of MHC class I antigens and are able to lyse fresh tumour cells without prior antigen exposition (Grimm *et al.*, 1983), activates T-cells to produce antigen specific cytolytic and antiproliferative responses against tumour cells (Smith, 1988), and induces the release of secondary cytokines such as interferon (INF) gamma and tumour necrosis factor (TNF) alfa from activated T-cells and macrophages (Stotter *et al.*, 1989).

INF alpha, a product of leucocytes, was initially identified through its antiviral activity (Isaacs & Lindenmann, 1957). INF alpha also has antitumour activity, either by direct antiproliferative effects on tumour cells (Fidler *et al.*, 1987), or indirectly by the induction of MHC class I or tumour associated antigens on tumour cells (Brunda *et al.*, 1987).

The therapeutic potential of both IL-2 and INF alpha as single agents has been studied in renal cell carcinoma and melanoma. Published clinical trials of INF alpha have reported response rates between 10-17% in renal cell carcinoma and melanoma (Hersey *et al.*, 1985; Quesada *et al.*, 1985). Phase II studies of IL-2 with or without LAK cells have reported tumour responses of 16-31% in renal cell carcinoma and 14-26% in melanoma (Rosenberg *et al.*, 1988; Fischer *et al.*, 1988; Stahel *et al.*, 1989; Dutcher *et al.*, 1989; Bar *et al.*, 1990), although at the cost of considerable acute toxicity.

With the aim to increase tumour response and/or reduce IL-2 induced toxicity, clinical studies are currently under way using IL-2 in combination with other cytokines or with cytostatic agents. Our decision to study IL-2 and INF alfa-2a was based on three factors: (1) Either agent alone has antitumour activity against melanoma or renal cell carcinoma, (2) in combination, these two agents have a synergistic antitumour effect in the mouse model (Brunda *et al.*, 1987) and (3) INF alfa-2a enhances the MHC class I and tumour associated antigen expression on the cell surface of tumour cells (Borden *et al.*, 1988) and thus may render them more susceptible for IL-2 induced cellular cytotoxicity.

We report here the clinical and immune modulatory effects of alternative weekly continuous infusion IL-2 and subcutaneous INF alfa-2a in 14 patients with renal cell carcinoma or melanoma, treated as part of a multicentre trial. Our findings suggest, that alternative weekly treatment at doses used here, results in an exhaustion of the lytic capacity of peripheral blood lymphocytes and an attenuation of secondary cytokine release.

### Materials and methods

### *Patient selection*

Eight patients with metastatic melanoma and six patients with advanced renal cell carcinoma were treated as part of a multicentre trial between December 1988 and October 1989. Selection criteria were measurable disease, prior nephrectomy in patients with renal cell carcinoma, no chemotherapy, hormonal therapy or radiotherapy within 4 weeks prior to study entry and no prior immunotherapy. Further eligibility criteria were a Karnofsky index of at least 60%, no clinical or radiological evidence of brain metastasis, no significant alterations in organ function, no endocrine disorders, and negative HIV and hepatitis serology. The protocol was approved by the institutional review board and all patients gave written informed consent.

### *Treatment*

IL-2 (Ro 23-6019) and INF alfa-2a (Ro 22-8181) were provided by Hoffman-LaRoche Basle, Switzerland and Nutley, NJ USA as a lyophilised powder in sterile vials. An intravenous drug delivery system (Port-A-Cath, Pharmacia AG, Dübendorf, Switzerland) was implanted in all patients prior

Correspondence: R.A. Stahel, M.D.
Received 6 September 1990; and in revised form 10 October 1990.

to start of treatment and IL-2 was given via a computerised ambulatory drug delivery pump (Pharmacia Deltec CADD-1 Model 5100 HF, Pharmacia AG, Dübendorf Switzerland). Treatment consisted in a 3 MU/m2 IL-2 as continuous infusion on days 1-4 and 6 MU/ms INF alfa-2a subcutaneously on days 1 and 4, both given on alternate weeks. Patients were given two cycles of treatment as inpatients and continued as outpatients, if initial treatment was well tolerated and no grade III toxicity was observed. Patients received novaminsulfone and paracetamol for fever, pethidine for rigors, metoclopropamide and domperidon for nausea and clemastin for erythema. Tumour response was assessed after four cycles of treatment, or earlier if deemed necessary by the investigator. Patients with stable disease or response were to be continued for nine more cycles or up to disease progression or patients intolerance.

*Toxicity and response criteria*

Patients were evaluated for toxicity daily during treatment and once during the 10 day resting period. Clinical toxicity was assessed according to WHO classification of Grade I-IV and tumour response was assessed according to established criteria as CR, PR, MR and PD (WHO Handbook, 1979).

*Immune modulatory parameters on peripheral blood mononuclear cells*

Immune modulatory parameters were measured as follows: Cycle 1, 14 patients; cycle 2, eight patients; cycle 3, 12 patients; and cycle 4, six patients. Fresh peripheral blood mononuclear cells (PMNC) were obtained from blood drawn through a central venous line into heparinised Vacutainer (Becton-Dickinson, Basle, Switzerland) glass tubes. A Ficoll (Seromed, Fakola AG, Basle, Switzerland) centrifugation was performed, cells were washed twice in Hank's balanced salt solution (HBSS), and resuspended $1 \times 10^6$ PMNC $ml^{-1}$ in HBSS. For phenotyping aliquots of 50 $\mu l$ were incubated for 30 min at room temperature in the dark with 5-20 $\mu l$ of monoclonal antibodies against the CD16 and CD56 antigens labelled with fluorescein isothiocyanate or phycoerythrin (Becton-Dickinson, Basle, Switzerland). After washing the PMNC were fixed with paraformaldehyde 0.5% in Ultracount (Becton-Dickinson, Basle, Switzerland) resulting in a final volume of 200 $\mu l$/assay. The direct immunofluorescence analysis was done on a EPICS Profile Analyser (Coulter Electronics, Instrumenten-Gesellschaft, Zürich, Switzerland).

Daudi and K562 cell lines were used for the assessment of LAK- and NK-activity, respectively. Target cells were labelled with 100 $\mu$Ci $^{51}$chromium $10^{-6}$ cells (Amersham, Rahn AG, Zürich, Switzerland) according to standard procedure. Ten thousand target cells/well were plated in a microtiter plate (Falcon, Inotech AG, Wohlen, Switzerland) in 50 $\mu l$ of complete medium consisting (CM) of RPMI 1640 (Flow Laboratories AG, Baar, Switzerland) supplemented with 10% foetal calf serum, 2 mM L-glutamine, 50 $\mu g$ $ml^{-1}$ streptomycin, 50 U $ml^{-1}$ penicillin and 100 U $ml^{-1}$ recombinant IL-2 (F. Hoffmann-La Roche & Co, Basle Switzerland). PMNC's were preincubated for 1 h ($1 \times 10^6$ cells $ml^{-1}$ at 37°C, 5% $CO_2$) in CM. Appropriate 1:2 dilutions of effector cells in 100 $\mu l$ CM were added to the target cells resulting in effector/target ratios ranging from 40:1 to 1.25:1. Spontaneous and maximal $^{51}$chromium-release was obtained by the addition of 100 $\mu l$ cell free CM and 0.1 molar hydrochloride respectively. Microtiter plates were incubated for 4 h (at 37°C, 5% $CO_2$), the supernatants harvested with a Skatron Harvester system (Tecnomara AG, Zürich, Switzerland) and counted (2 min/probe) with a Gamma-Counter (LKB Clinigamma, Pharmacia AG, Dübendorf, Switzerland). All tests were done in quadruplicate. Specific tumour cell lysis was calculated according to the formula: (experimental cpm-spont. cpm)/(max. cpm-spont. cpm) × 100. Lytic units (LU) per ml blood were calculated based on the E/T-ratio at the intercept of 20% specific lysis of 5,000 target cells.

*Determination of secondary cytokines*

Five ml of venous blood was drawn on days 2-4 of cycles 1-4 in seven consecutive patients through a central venous line into Vacutainer glass tubes (Becton-Dickinson, Basle, Switzerland). After centrifugation with 1,000 r.p.m. for 10 min, supernatant was aliquoted and stored at -20°C. TNF alpha and INF gamma serum levels were determined by the immunoradiometric assay kit (JRE-Medgenix AG-B6620, Fleurus, Belgium).

*Statistical analysis*

All clinical and laboratory data were evaluated on a personal computer using symphony spreadsheets. The paired two sided *t*-test was used for comparison of immune modulatory parameters between days 1 and 8 of cycles 1-4.

### Results

*Patient and treatment summary*

Eight patients with melanoma and six patients with renal cell carcinoma with a mean age of 47 (range 32-60 years) and a mean Karnofsky index of 90% (range 80-100%) were entered into the study (Table I). All patients had surgery (either nephrectomy or excision of the primary tumour or metastasis). Four patients received radiotherapy, three chemotherapy and one hormone therapy prior to entry into the study.

The 14 patients received a total of 60 cycles of treatment, of which 20 were administered as outpatients (Figure 1). The following dose adjustments were made: Patients No 4M and 2R had a 50% dose reduction after the first and the fourth cycle, respectively, because of grade III hematological and gastrointestinal toxicity. Patient 3M had a cystic degeneration of a subcutaneous metastasis during treatment which required multiple aspirations of fluid and finally had to be surgically removed after three cycles of treatment. In patients 5M and 6M treatment was discontinued after four cycles despite the achievement of a partial response, because of *Staphylococcus aureus* sepsis and patient refusal, respectively.

*Toxicity*

The highest grade of clinical toxicity per patient recorded during treatment is summarised in Table II. All patients had systemic symptoms consisting in fever, malaise and chills. Twelve patients had a desquamative or maculopapular erythema. Gastrointestinal toxicity consisted in loss of appetite, nausea, vomiting, diarrhoea and mucositis. One patient with pre-existing paroxysmal supraventricular tachycardia had an episode of supraventricular tachycardia requiring intravenous verapamil. All patients had varying degrees of capillary leak syndrome with a mean weight gain of 2.5% of entry weight, hypotension, pericardial and pleural effusion and exertional dyspnea. A rise in serum creatinine was only observed in patients with prior nephrectomy. CNS toxicity consisted in nightmares and delusions. Two major infections were observed with *Staphylococcus aureus* sepsis originating from a central venous line. Hematological toxicity consisted in anaemia (four patients with Grade I, seven patients with Grade II) and leucopenia and thrombopenia (one patient Grade III). Five of 14 patients (36%) developed a self-limiting autoimmune thyroiditis with a short hyperthyroid phase followed by a prolonged hypothyroid phase. Fine needle aspirations, done in three patients showed a lymphohistiocytic infiltrate with HLA-DR expression on thyroid epithelial cells (Pichert *et al.*, 1990). Long term toxicity was moderate with an average weight loss of 5% of entry value and an average decrease of 10% in the Karnofsky index.

**Table I** Patient characteristics and antitumour response

| *Patient no/ diagnosis* | *Age* | *Sex* | *Site of metastasis* | *No. of cycles* | *Response* | *Duration* | *Thyroiditis* |
|---|---|---|---|---|---|---|---|
| 1R | 45 | M | Subcutaneous, lung, liver | 4 | PD | | Yes |
| 2R | 58 | M | Medistinal, lung, soft tissue | 10 | NC | 3 months | No |
| 3R | 49 | F | Lymph nodes | 4 | PD | | No |
| 4R | 45 | M | Mediastinal | 4 | PD | | Yes |
| 5R | 59 | M | Local recurrence, lung, soft tissue | 4 | PD | | No |
| 6R | 40 | M | Lung | 1 | PD | died | No |
| 1M | 60 | M | Mediastinal, lung, subcutaneous | 4 | PD | | No |
| 2M | 43 | F | Lung | 8 | MR | 2 months | Yes |
| 3M | 49 | F | Subcutaneous | 3 | MR | 3 months | Yes |
| 4M | 24 | F | Local recurrence, lung | 3 | PD | | No |
| 5M | 49 | M | Lymph node | 4 | PR | 7 months | Yes |
| 6M | 49 | F | Lymph nodes, lung, liver, soft tissue | 5 | PR | 1 month | No |
| 7M | 37 | M | Lung, liver, lymph nodes, subcutaneous | 3 | PD | | No |
| 8M | 36 | M | Lung, subcutaneous | 3 | PD | | No |

R = renal cell carcinoma, M = melanoma.

**Figure 1** Treatment summary. Sixty cycles of IL-2 and INF alfa-2a were given to 14 patients. Twenty cycles could be administered on an outpatient basis.

**Table II** Clinical toxicity: $n = 14$ patients

| *Side effect* | *Grade I* | *Grade II* | *Grade III* | *Grade IV* |
|---|---|---|---|---|
| Constitutional | – | 9 | 5 | – |
| Skin | 1 | 11 | – | – |
| GJ | – | 11 | 3 | – |
| Heart | 1 | – | – | – |
| Lung | – | 6 | 3 | – |
| Blood pressure | 2 | 4 | 4 | – |
| Renal | 5 | – | – | – |
| CNS | 1 | – | – | – |
| Infections | – | – | – | 2 |
| Hematological | 4 | 7 | 1 | – |

Constitutional: Fever, malaise, chills, arthralgias; Skin: Erythema with pruritus; GJ: Nausea and vomiting; Heart: supraventricular tachycardia; Lung: dyspnea, pleural effusion; Renal: elevated serum creatinine; Blood pressure: hypotension; CNS: nightmares and delusions; Infections: sepsis with staph. aureus; Hematologic: anemia, leucopenia and thombopenia.

*Antitumour effect*

No responses were seen in renal cell carcinoma. Two patients with melanoma, one with regional lymph node metastasis and one with extensive visceral metastasis (Figure 2) had a partial response, and two patients had a minor response (Table I). The responses lasted from 1 to 7 months.

**Figure 2** Abdominal CT scan of patient No. 6M, showing a right mesenterial metastasis: **a**, before treatment and **b**, after five cycles of IL-2 and INF alfa-2a.

*Immune modulatory effects*

Lymphocyte subset determination and measurement of NK- and LAK-activity were done on days 1 and 8 of cycles 1–4 and INF gamma and TNF alfa serum levels were determined on days 2–4 of cycles 1–4.

During every treatment cycle, lymphopenia was observed on day 4 followed by a rebound lymphocytosis on day 8 (Figure 3). CD16- and CD56-positive cells as indicators for activated lymphocytes and NK-cells increased 2.5-fold ($P<0.01$) and 2-fold ($P<0.01$) during cycle 1, and 1.8-fold (n.s.) or 2.6-fold respectively ($P<0.05$) during cycle 4 (Figure 4).

In contrast to the consistent treatment induced increase of CD16 and CD56 pos. cells over the first four cycles; LAK- and NK-activity increased during the first two cycles only, plateaued during the third cycle and even decreased (n.s.) during the fourth cycle (Figure 5).

Examination of treatment induced release of secondary cytokines showed no significant changes in TNF alpha levels while INF gamma levels decreased with successive cycles with a significant difference between the first and the third cycle of treatment ($P<0.05$). (Figure 6).

**Figure 3** Mean values and standard deviation of circulating lymphocytes, measured on day 1, 4 and 8 of cycles 1–4.

**Figure 4** Mean values and standard deviation of circulating **a**, CD16 and **b**, CD56-positive cells, measured on day 1 and 8 of cycles 1–4.

### Discussion

Our regimen of IL-2 3 MU/ms continuous infusion on days 1–4 and INF alfa-2a subcutaneously on day 1 and 4, given on alternative weeks, had only moderate toxicity. The initial concern of cumulative weight loss and decrease of Karnofsky index by repetitive cycles of therapy was not substantiated. The most frequent clinical toxicities were constitutional symptoms, erythema with pruritus, nausea and vomiting, and varying degrees of capillary leak syndrome. The first two cycles of treatment were given in the hospital and in six of 14 patients further treatment could be administered on an out-patient basis. None of the patients required intensive care facilities. The only Grade IV toxicity observed was catheter related sepsis with *Staphylococcus aureus*, which occurred in two patients. Recently, it has been reported that IL-2 therapy is associated with an increased risk of bacterial sepsis, probably due to an IL-2 induced defect of neutrophil chemotaxis (Klempner *et al.*, 1990). Prophylactic antibiotic therapy might thus be of advantage in further studies using IL-2.

We have previously described a self-limiting form of auto-immune thyroiditis with expression of HLA-DR on thyrocytes in patients undergoing combined IL-2 and INF alfa-2a therapy (Pichert *et al.*, 1990). The overall incidence of auto-immune thyroiditis in the 14 patients reported here was 36%. An association of thyroiditis with tumour response has been suggested for patients treated with IL-2 and LAK-cells (Atkins *et al.*, 1988). Our findings of tumour response in three of five patients with thyroiditis *vs* one of nine euthyroid patients support this hypothesis. This phenomenon could be due to the fact that HLA-DR antigen expression and auto-reactive T-cell clones might contribute to the development of thyroiditis as well as antitumour response.

Final data on response rates with our regimen of IL-2 and INF alfa-2a will only be available when all data from the multicenter trial has been analysed. In part of the study, tumour response was only seen in patients with melanoma (two partial and two minor responses). Using the combination of IL-2 in INF alfa-2a, we and others found little effectivity in renal cell carcinoma (Lee *et al.*, 1989). These findings are in contrast to reports by Rosenberg *et al.*, 1989, who reported response rates of 17–41% in both melanoma and renal cell carcinoma with escalating doses of IL-2 (3–13.5 MU/m2 per day) and INF alfa-2a (9–18 MU/ms per day), suggesting a possible dose response relationship for the combination of IL-2 and INF alfa-2a.

Serial measurement of immune modulatory parameters gave unexpected findings. While there was a significant rebound lymphocytosis with each treatment cycle, the functional response of peripheral blood lymphocytes measured by NK- and LAK-activity increased only during the first two cycles of treatment. During the third cycle the functional response plateaued and during the fourth cycle NK- and LAK-activity even decreased. These findings were paralleled by a successive decline of treatment-induced INF gamma response, presumably the product of activated T-cells, while the TNF alpha response, mainly a product of macrophages was stable or did increase slightly. The decline in treatment-induced lytic capacity of peripheral blood lymphocytes and serum levels of INF gamma suggest that there is an exhaustion of functional capacity of peripheral blood lymphocytes with repetitive alternative weekly treatment with IL-2 and INF alfa-2a. Earlier reports have described that repetitive doses of human leucocyte interferon in patients with melanoma can lead to a decrease in NK-activity, which may be due

**Figure 5** Mean values and standard deviation of **a**, NK and **b**, LAK activity, measured on day 1 and 8 of cycles 1–4.

**Figure 6** Induction of **a**, INF gamma and **b**, TNF alpha during treatment. Mean serum levels with standard deviation measured on days 2–4 of cycles 1–4.

to a therapy-acquired unresponsiveness of lymphocytes (Maluish *et al.*, 1983; Golub *et al.*, 1982). An alternative explanation for the attenuation of functional lytic response might be the schedule dependency of the immune modulatory effects caused by combined IL-2 and INF alfa-2a. In animal models a synergistic effect on LAK activity has been seen when INF was given before or after IL-2, while an inhibitory effect was seen with concomitant administration of the two agents (Tokuda *et al.*, 1989; Brunda *et al.*, 1986). Thus the timing of INF alfa-2a application when given in combination with IL-2 might be important and these results should be taken into account in designing further studies.

We thank Heidi Ernst and the nursing staff of the Division of Oncology for their contribution to the study.

### References

ATKINS, M.B., MIER, J.W., PARKINSON, D.R. & 4 others (1988). Hyopthyroidism after treatment with interleukin-2 and lymphokine-activated killer cells. *New Engl. J. Med.*, **318,** 1557.

BAR, M.H., SZOL, M., ATKINS, M.B. & 12 others (1990). Metastatic malignant melanoma treated with combined bolus and continuous infusion interleukin-2 and lymphokine activated killer cells. *J. Clin. Oncol.*, **8,** 1138.

BORDEN, E.C. (1988). Augmented tumor-associated antigen expression by interferon. *J. Natl Cancer Inst.*, **80,** 8.

BRUNDA, M.J., BELLANTONI, D. & SULICH, V. (1987). *In vivo* antitumor activity of combinations of interferon-a and interleukin-2 in a murine model. Correlation of efficacy with the induction cytotoxic cells resembling natural killer cells. *Int. J. Cancer*, **40,** 365.

BRUNDA, M.J., TARNOFSKI, D. & DAVATELIS, V. (1986). Interaction of recombinant interferons with recombinant interleukin-2: differential effects on natural killer cell activity and interleukin-2 activated killer cells. *Int. J. Cancer*, **37,** 787.

DUTCHER, J.P., CREEKMORE, S., WEISS, G.R. & 11 others (1989). A phase II study of interleukin-2 and lymphokine activated killer cells in patients with metastatic melanoma. *J. Clin. Oncol.*, **7,** 477.

FIDLER, I.J., HEICAPPELL, R., SAIKI, I., GRUTTER, M.G., HORISBERGER, M.A. & NUESCH, J. (1987). Direct antiproliferative effects of recombinant human interferon-a. B/D hybrids on human tumor cell lines. *Cancer Res.*, **47,** 2020.

FISHER, R.I., COLTMAN, C.A., DORSHOW, J.H. & 11 others (1988). Metastatic renal cancer treated with interleukin-2 and lymphokine activated killer cells. *Ann. Intern. Med.*, **108,** 518.

GOLUB, S.H., D'AMORE, P. & RAINEY, M. (1982). Systemic administration of human leucocyte interferon to melanoma patients. II. Cellular events associated with changes in natural killer cytotoxicity. *J. Natl Cancer Inst.*, **68,** 711.

GRIMM, E.A., ROBB, R.J., ROTH, J.A. & 4 others (1983). Lymphokine activated killer cell (LAK) phenomenon III. Evidence that IL-2 alone is sufficient for direct activation of PBL into LAK. *J. Exp. Med.*, **158,** 1356.

HERSEY, P., HASIC, E., MACDONALD, M. & 5 others (1985). Effects of recombinant leucocyte interferon (rIFN-alfa A) on tumor growth and immune responses in patients with metastatic melanoma. *Br. J. Cancer*, **51,** 815.

ISAACS, A. & LINDENMANN, J. (1957). Virus interference. 1. The interferons. *Proc. R. Soc. Lond. Biol. Sci.*, **147,** 258.

KLEMPNER, M.S., NORING, R., MIER, J.W. & ATKINS, M.B. (1990). An acquired chemotactic defect in neutrophils from patients receiving interleukin-2 immunotherapy. *New Engl. J. Med.*, **322**, 959.

LEE, K.H., TALPAZ, M., ROTHBERG, J.M. & 6 others (1989). Concommitant administration of recombinant human interleukin-2 and recombinant interferon alfa-2a in cancer patients: a phase II study. *J. Clin. Oncol.*, **7**, 1726.

MALUISH, A.E., ORTALDO, J.R., CONLON, J.C. & 6 others (1983). Depression of natural killer cytotoxicity after *in vivo* administration of recombinant leucocyte interferon. *J. Immunol.*, **131**, 1.

MORGAN, D.A., RUSCETTI, F.W. & GALLO, R. (1978). Selective *in vitro* growth of T-lymphocytes from normal human bone marrow. *Science*, **193**, 1007.

PICHERT, G., JOST, L.M., ZÖBELI, L., ODERMATT, B., PEDIO, G. & STAHEL, R.A. (1990). Thyroiditis after treatment with interleukin-2 and interferon alfa-2a. *Br. J. Cancer*, **62**, 100.

QUESADA, J.R., RIOS, A., SWANSON, D., KURZROCK, R. & GUTTERMAN, J.U. (1985). Antitumor activity of recombinant-derived interferon alfa in metastatic renal cell carcinoma. *J. Clin. Oncol.*, **3**, 1522.

ROSENBERG, S.A., LOTZE, M.T. & MULE, J.J. (1988). New approaches to the immunotherapy of cancer using interleukin-2. *Ann. Int. Med.*, **108**, 853.

ROSENBERG, S.A., LOTZE, M.A., YANG, J.C. & 7 others (1989). Combination therapy with interleukin-2 and alfa-interferon for the treatment of patients with advanced cancer. *J. Clin. Oncol.*, **7**, 1863.

SMITH, K.A. (1988). Interleukin-2: inception, impact, and implications. *Science*, **240**, 1169.

STAHEL, R.A., SCULIER, J.P., JOST, L.M. & 7 others (1989). Tolerance and effectiveness of recombinant interleukin-2 (r-met HU IL-2 (ala-125)) and lymphokine-activated killer cells in patients with metastatic solid tumors. *Eur. J. Cancer Clin. Oncol.*, **25**, 965.

STOTTER, H., WIEBKE, E.A., TOMITA, S. & 4 others (1989). Cytokines alter target cell susceptibility to lysis. *J. Immunol.*, **142**, 1767.

TANIGUCHI, T., MATSUI, H., FUJITA, T. & 5 others (1983). Structure and expression of a cloned cDNA for human interleukin-2. *Nature*, **302**, 305.

TOKUDA, Y., EBINA, N. & GOLUB, S.H. (1989). The inhibitory effect of human interferon alfa on the generation of lymphokine-activated killer activity. *Cancer Immunol. Immunother.*, **30**, 205.

WHO GENEVA (1979). *WHO Handbook for reporting results of cancer treatment*. WHO offset publication No. 48, WHO: Geneva, 1979.